# The Effects of Sensory Electrical Stimulation and Local Vibration on Motor Learning and Motor Function

**DOI:** 10.1298/ptr.R0036

**Published:** 2025-03-13

**Authors:** Wan-Yan TSENG, I-Hsiang TSENG, Li-Wei CHOU

**Affiliations:** Department of Physical Therapy and Assistive Technology, National Yang Ming Chiao Tung University, Taiwan

**Keywords:** Local vibration, Motor function, Motor learning, Neuromuscular control, Rehabilitation, Sensory electrical stimulation

## Abstract

Sensory afferent inputs play a crucial role in neuromuscular control. Enhancing sensory input through electrical or mechanical stimulation of the limbs may improve motor function and facilitate motor learning. This scoping review synthesizes literature investigating the effects of sensory electrical stimulation (SES) and local vibration (LV) on motor function and learning in both healthy individuals and those with musculoskeletal or neurological disorders. The findings suggest that SES can enhance motor learning and improve motor function. Furthermore, its efficacy is maximized when combined with rehabilitation programs and motor training rather than being used as a stand-alone intervention. Similarly, LV applied to muscle or tendon regions enhances proprioceptive input, thereby improving motor control and learning. The clinical benefits of LV, like those of SES, can be augmented by incorporating it into motor training regimens. Future research should focus on optimizing stimulation parameters and determining the most effective integration strategies for rehabilitation programs to maximize therapeutic outcomes.

## Introduction

Motor learning and control are essential components of daily activities^1)^. Sensory afferent inputs are fundamental for executing motor functions^2,3)^, and their impairment often results in motor deficits^4,5)^. Motor learning is broadly defined as “a change in motor performance induced by practice”^6)^ and is essential for acquiring new motor skills or relearning movements following physiological or pathological changes, such as aging, disease, or injury. Neural plasticity, a key aspect of motor learning^7,8)^, is facilitated by sensory inputs that modulate central nervous system activity^9,10)^. Traditionally, motor learning is achieved through repetitive practice, during which sensory feedback and sensorimotor cortical integration refine motor programs, reducing error rates while improving speed and accuracy^9)^. This approach is commonly employed in motor relearning for patients with motor impairments due to central nervous system disorders, musculoskeletal injuries, or post-surgical conditions. However, it has inherent limitations. For instance, in the acute post-stroke phase, motor impairments caused by abnormal muscle tension or spasticity may hinder repetitive motor practice. Similarly, postoperative patients may experience motor disabilities due to pain or surgical wounds, preventing active participation in motor training^11)^. Given these challenges, alternative therapeutic approaches, such as motor imagery, mental practice, sensory electrical stimulation, and local muscle vibration, have been developed to enhance motor learning and function^12–15)^.

During motor learning, the sensory cortex receives multiple sensory inputs (e.g., visual, cutaneous, and proprioceptive), which provide essential information necessary for motor system adjustments and corrections. This process reduces discrepancies between intended motor programs and actual output^1,16)^. Proprioception, in particular, offers direct feedback on movement execution, including joint angles, muscle length, and limb position. Therefore, adequate sensory input and sensory cortical activation are critical for motor learning^17)^. Conversely, impaired sensory systems lead to immediate motor function decline and hinder motor relearning^16)^, negatively affecting recovery in patient populations^18,19)^.

Given the essential role of sensory input in motor learning, researchers have proposed that augmenting sensory input may enhance motor relearning and improve motor function, including muscle strength, gait, and balance, by inducing neural adaptation. Providing additional sensory input can enhance the plasticity and excitability of the sensorimotor cortex and neural pathways, thereby improving motor learning^20,21)^. Based on this theory, sensory stimulation has been employed as a neural modulation strategy to enhance motor function. Two common forms of peripheral sensory input include electrical stimulation and vibration. Currently, there is a lack of comprehensive reviews on how different sensory inputs facilitate motor learning. Although these techniques have shown promise individually, their effects vary based on application parameters, such as duration and frequency, and may depend on specific patient motor deficits. This review focuses on the benefits of electrical stimulation and vibration as sensory stimuli, discussing their underlying mechanisms and suggesting clinical application parameters for optimizing motor learning and function.

## Sensory Electrical Stimulation

Sensory electric stimulation (SES) has been extensively studied for its role in enhancing motor learning and recovery, particularly in individuals with neurological impairments such as stroke. SES is the most commonly used form of electrical stimulation, wherein electrical impulses are applied to peripheral areas with intensities sufficient to activate cutaneous nerve fibers, primarily Aβ and Aδ fibers, which are located superficially in the stimulation area. This stimulation elicits sensations such as tingling or prickling, resembling numerous tiny sharp objects lightly poking the skin. SES can be administered at two primary sites: directly to the targeted body area or the nerve trunk (axon) that innervates the targeted area. For instance, if SES is intended to enhance hand function, some studies have applied stimulation directly to the muscle belly, while others have stimulated the median nerve, which innervates the hand muscles. The latter approach activates the entire dermatome, potentially affecting a broader area compared with muscle-specific stimulation.

SES has been shown to enhance proprioceptive feedback, improve motor execution accuracy, reduce sensorimotor uncertainty, and facilitate motor learning^22)^. Studies have demonstrated its benefits at various stages of motor learning. For example, Veldman et al.^23)^ conducted an experiment in which participants received either 20 min of real SES or a control intervention. They observed changes in visuomotor task performance and cortical adaptations using electroencephalography. Their findings indicated that visuomotor task performance improved immediately after SES and on Day 2, suggesting that SES enhances motor skill acquisition and consolidation, likely by increasing sensorimotor cortex activity and connectivity.

Veldman et al.^14)^ further examined whether the duration of SES influences motor learning. Participants were randomly assigned to receive 20, 40, or 60 min of real SES or sham stimulation. Their study revealed that 20 min of SES enhanced visuomotor task performance on Days 2 and 7, whereas 60 min of SES improved performance on Day 7. Additional research demonstrated similar benefits of SES on motor learning by stimulating two (ulnar and median) or three (ulnar, median, and radial) nerves in the upper limb^24)^.

Moreover, SES exerts both direct and cross effects on motor learning. By comparing visuomotor task performance following motor practice, SES alone, or SES combined with motor practice, researchers found that SES alone could induce motor learning effects comparable to those of motor practice. Additionally, these learning effects transferred to the non-stimulated hand, indicating broader neurophysiological impacts beyond the stimulation site^20)^.

SES has also been shown to enhance motor control, particularly by reducing reaction times and increasing response vigor. A study using high-frequency electrical stimulation demonstrated that SES can effectively facilitate the early release of motor actions, with increased stimulation intensity correlating with shorter reaction times and greater response vigor^25)^. Furthermore, SES can target either muscle or skin afferents, both of which contribute to the facilitation of motor actions. These findings highlight SES’s potential for enhancing immediate sensory input to improve motor control and performance.

The effects of SES on patient populations, particularly individuals recovering from stroke, have been investigated in various clinical studies. Systemic reviews have been conducted to evaluate the clinical benefits of SES^26–28)^. However, the results of these trials have been mixed. Some studies reported low to moderate evidence supporting the effectiveness of SES in improving sensorimotor function, whereas others found no significant difference between SES and placebo or standard care. These findings suggest that SES alone may be insufficient for addressing severe motor deficits or improving complex motor tasks, such as gait, which require the integration of balance, coordination, and muscle control.

The limitations of SES as a stand-alone intervention underscore the need for more targeted approaches, such as combining SES with task-specific gait training, to more effectively address multifaceted motor demands. A systematic review and meta-analysis found that incorporating SES into rehabilitation programs enhances motor recovery post-stroke^29)^. This study analyzed 11 clinical trials and controlled studies in which SES was integrated into routine rehabilitation programs for individuals with chronic stroke. Compared with routine rehabilitation alone, the addition of SES improved motor function; however, not all studies reported positive clinical benefits. How SES was combined with rehabilitation appears to be a key factor influencing outcomes.

For example, one study demonstrated that applying SES before each motor training session enhanced neuromuscular control and motor function in individuals with chronic stroke^30)^. In this study, participants underwent an intervention twice per week for 8 weeks. During each session, SES was applied to the median nerve of the paretic limb for 30 min, followed immediately by 30 min of hand-functional training. The results indicated that, compared with motor training alone, SES combined with motor training led to greater improvements in motor function, as measured by Fugl–Meyer Assessment scores. These findings highlight the importance of combining SES with motor training, demonstrating its potential to enhance rehabilitation outcomes. Administering SES before training sessions may improve sensory feedback, priming the nervous system to reinforce coordinated neuromuscular patterns and control. This, in turn, can lead to significant improvements in mobility and independence for individuals with motor impairments. Another promising approach is the application of SES immediately after motor training sessions, as it may reinforce motor learning and reconsolidation^31,32)^, thereby enhancing movement patterns. While combining SES with motor training offers substantial clinical benefits, further research is needed to determine the optimal timing and duration of SES application in rehabilitation settings to maximize its effectiveness.

## Local Vibration

Local vibration (LV) provides mechanical stimulation to the neuromuscular system, triggering physiological responses that have been extensively reviewed^33)^. When applied to muscle or tendon regions, LV activates proprioceptors, such as muscle spindles, enhancing sensory feedback and facilitating sensorimotor integration. The following section will explore the immediate neurophysiological responses to LV. Additionally, when LV is administered repetitively as part of an intervention or training program, it generates continuous sensory afferent input, inducing neural plasticity in the sensorimotor pathways. This integration of proprioceptive input into the central nervous system enhances motor planning and learning^17,21,34)^.

LV at frequencies ranging from 80 to 120 Hz elicits distinct neural adaptations by stimulating muscle spindle receptors and Ia afferents^35–37)^. Studies have shown that LV applied to skeletal muscle generates a tonic vibration reflex, leading to various neurophysiological responses in the central nervous system. First, continuous proprioceptive input creates an illusion of limb movement^38,39)^. Additionally, LV decreases motoneuron excitability at the spinal level, as evidenced by a reduced H-reflex amplitude^40,41)^. Recent research^42)^ has further demonstrated that prolonged LV (30 min) can decrease motor neuron excitability while increasing cortical excitability, as indicated by an enhanced motor-evoked potential. These findings suggest that LV induces a reorganization in corticomuscular activation, wherein reduced spinal excitability is counterbalanced by increased motor cortex activity.

Short-term LV (lasting seconds to minutes) can facilitate motor skill acquisition and retention by influencing corticocerebellar processing. In a study conducted by Pettorossi et al.^43)^, the effects of neck vibration stimuli ranging from 5 to 100 Hz were compared. The findings revealed that vibrations exceeding 80 Hz and applied for more than 8 min enhanced individuals’ perception of whole-body rotation. Another study using the same frequency (80 Hz) but a shorter duration (30 s) found that increasing proprioceptive signal uncertainty improves motor coordination and control, leading to significant enhancements in motor performance, such as reaction time, within a short period^44)^. However, the effects of LV on neuromuscular excitability and motor function are transient. Evidence suggests that after vibration stimulation, the H-reflex and reciprocal spinal inhibition return to baseline within 60 min^45)^.

From a clinical perspective, LV has been shown to promote motor recovery in patients with chronic conditions by alleviating localized functional impairments and activating compensatory mechanisms. However, as previously mentioned, these changes typically require prolonged stimulation over several weeks to months^21,46)^. Therefore, extending the duration of LV interventions or combining them with exercise training may enhance their long-term effects on neuromuscular excitability and motor recovery.

Long-term LV training typically incorporates LV alongside strength or motor training for several weeks. In general, LV training has demonstrated promising clinical benefits. For instance, a study involving 300 Hz LV training over 12 weeks in elderly individuals with sarcopenia reported enhanced muscle strength and increased fast-twitch fiber content (Myosin Heavy Chain 2X)^47)^, suggesting that LV training may counteract muscle atrophy associated with aging. Additionally, randomized controlled trials have shown that voluntary leg muscle contractions at 50 Hz for 14 days^48)^ and at 100 Hz for 8 weeks^49)^ resulted in strength gains. Notably, the 8-week LV training also induced strength improvements in the untrained leg. Furthermore, a 4-week LV training study confirmed that these effects were consistent across both young and elderly populations^49)^.

LV training has also been shown to enhance motor function. A review by Holmes et al.^50)^ suggests that prolonged suprathreshold vibration induces lasting improvements in balance among older adults. For example, a 4-week localized vibration study (120 Hz), administered three times per week, improved gait parameters in patients with diabetic peripheral neuropathy^51)^. Another study utilizing a wearable vibration device (100 Hz) embedded in compression pants demonstrated significant improvements in postural stability in older adults^52)^. Additionally, a study combining strength training with LV found that after 4 weeks of training, LV significantly increased isometric maximal strength compared with the limb that did not receive LV^53)^, underscoring the additional clinical benefits of LV when integrated with motor training.

LV training has demonstrated substantial benefits for sensory and motor function by promoting cortical reorganization and enhancing cortical activation. These neural adaptations improve proprioceptive discrimination, motor execution efficiency, and performance consistency. Such benefits are particularly valuable in rehabilitation settings, where LV training has been shown to enhance motor control across diverse patient populations. These findings emphasize the versatility and efficacy of LV in promoting motor function, recovery, and overall functional performance. Given the variability in vibration parameters and protocols across studies, establishing a standardized LV training regimen remains challenging. However, based on existing evidence, training programs should incorporate higher vibration frequencies and extend intervention duration to a minimum of 4 weeks, with three training sessions per week, to yield positive clinical outcomes.

## Conclusion

This review examined studies that utilized electrical stimulation and LV to modulate the neuromuscular system, promote motor learning, and improve motor function. Both electrical stimulation and LV have demonstrated potential in enhancing motor learning and recovery, particularly in individuals with neurological impairments such as stroke. The benefits of electrical stimulation and LV are most pronounced when combined with motor training, as demonstrated in recent studies that highlight improvements in neuromuscular control and motor performance. Future research should focus on optimizing the integration of electrical stimulation and LV with motor training, particularly in terms of timing and intervention duration, to maximize therapeutic outcomes. Additionally, exploring the combination of electrical stimulation and LV with task-specific training, particularly for complex motor tasks such as gait, could provide valuable insights into improving mobility and functional outcomes in individuals with motor impairments.

## Funding

This work was partially supported by a grant from the National Science and Technology Council, Taiwan (Grant number: NSTC 113-2221-E-A49-041).

## Conflicts of Interest

The authors declare no conflicts of interest associated with this manuscript.

## References

[1] Halsband U Lange RK: Motor learning in man: a review of functional and clinical studies. J Physiol Paris. 2006; 99: 414–424. 16730432 10.1016/j.jphysparis.2006.03.007

[2] Gentilucci M Toni I, *et al.*: Tactile input of the hand and the control of reaching to grasp movements. Exp Brain Res. 1997; 114: 130–137. 9125458 10.1007/pl00005612

[3] Rabin E Gordon AM: Tactile feedback contributes to consistency of finger movements during typing. Exp Brain Res. 2004; 155: 362–369. 14689143 10.1007/s00221-003-1736-6

[4] Rothwell JC Traub M, *et al.*: Manual motor performance in a deafferented man. Brain. 1982; 105: 515–542. 6286035 10.1093/brain/105.3.515

[5] Sanes JN Mauritz K-H, *et al.*: Motor deficits in patients with large-fiber sensory neuropathy. Proc Natl Acad Sci USA. 1984; 81: 979–982. 6322181 10.1073/pnas.81.3.979 PMC344963

[6] Yadav G Duque J: Reflecting on what is “skill” in human motor skill learning. Front Hum Neurosci. 2023; 17: 1117889. 37484917 10.3389/fnhum.2023.1117889 PMC10356990

[7] Dayan E Cohen LG: Neuroplasticity subserving motor skill learning. Neuron. 2011; 72: 443–454. 22078504 10.1016/j.neuron.2011.10.008 PMC3217208

[8] Papale AE Hooks BM: Circuit changes in motor cortex during motor skill learning. Neuroscience. 2018; 368: 283–297. 28918262 10.1016/j.neuroscience.2017.09.010 PMC5762136

[9] Mirdamadi JL Block HJ: Somatosensory changes associated with motor skill learning. J Neurophysiol. 2020; 123: 1052–1062. 31995429 10.1152/jn.00497.2019

[10] Makino H Hwang EJ, *et al.*: Circuit mechanisms of sensorimotor learning. Neuron. 2016; 92: 705–721. 27883902 10.1016/j.neuron.2016.10.029 PMC5131723

[11] Lephart SM Pincivero DM, *et al.*: Proprioception of the ankle and knee. Sports Med. 1998; 25: 149–155. 9554026 10.2165/00007256-199825030-00002

[12] Carrasco DG Cantalapiedra JA: Effectiveness of motor imagery or mental practice in functional recovery after stroke: a systematic review. Neurologia (Engl ed). 2016; 31: 43–52. 10.1016/j.nrl.2013.02.003 23601759

[13] Stenekes MW Geertzen JH, *et al.*: Effects of motor imagery on hand function during immobilization after flexor tendon repair. Arch Phys Med Rehabil. 2009; 90: 553–559. 19345768 10.1016/j.apmr.2008.10.029

[14] Veldman MP Zijdewind I, *et al.*: Motor skill acquisition and retention after somatosensory electrical stimulation in healthy humans. Front Hum Neurosci. 2016; 10: 115. 27014043 10.3389/fnhum.2016.00115 PMC4792880

[15] Wang L Wang S, *et al.*: Effectiveness and electrophysiological mechanisms of focal vibration on upper limb motor dysfunction in patients with subacute stroke: a randomized controlled trial. Brain Res. 2023; 1809: 148353. 36990135 10.1016/j.brainres.2023.148353

[16] Huang Y Zhang X, *et al.*: Involvement of primary somatosensory cortex in motor learning and task execution. Neurosci Lett. 2024; 828: 137753. 38554843 10.1016/j.neulet.2024.137753

[17] Rosenkranz K Rothwell JC: Modulation of proprioceptive integration in the motor cortex shapes human motor learning. J Neurosci. 2012; 32: 9000–9006. 22745499 10.1523/JNEUROSCI.0120-12.2012 PMC6622352

[18] Nudo RJ Friel KM, *et al.*: Role of sensory deficits in motor impairments after injury to primary motor cortex. Neuropharmacology. 2000; 39: 733–742. 10699440 10.1016/s0028-3908(99)00254-3

[19] Kessner SS Bingel U, *et al.*: Somatosensory deficits after stroke: a scoping review. Top Stroke Rehabil. 2016; 23: 136–146. 27078117 10.1080/10749357.2015.1116822

[20] Veldman MP Zijdewind I, *et al.*: Direct and crossed effects of somatosensory electrical stimulation on motor learning and neuronal plasticity in humans. Eur J Appl Physiol. 2015; 115: 2505–2519. 26335625 10.1007/s00421-015-3248-z PMC4635177

[21] Filippi GM Rodio A, *et al.*: Plastic changes induced by muscle focal vibration: a possible mechanism for long-term motor improvements. Front Neurosci. 2023; 17: 1112232. 36908788 10.3389/fnins.2023.1112232 PMC9992721

[22] Whittier TT Weller ZD, *et al.*: I can step clearly now, the TENS is on: transcutaneous electric nerve stimulation decreases sensorimotor uncertainty during stepping movements. Sens. 2022; 22: 5442. 10.3390/s22145442 PMC9317326 35891122

[23] Veldman MP Maurits NM, *et al.*: Somatosensory electrical stimulation improves skill acquisition, consolidation, and transfer by increasing sensorimotor activity and connectivity. J Neurophysiol. 2018; 120: 281–290. 29641307 10.1152/jn.00860.2017

[24] Sorinola IO Bateman RW, *et al.*: Effect of somatosensory stimulation of two and three nerves on upper limb function in healthy individuals. Physiother Res Int. 2012; 17: 74–79. 21748825 10.1002/pri.515

[25] Marinovic W Milford M, *et al.*: The facilitation of motor actions by acoustic and electric stimulation. Psychophysiology. 2015; 52: 1698–1710. 26338375 10.1111/psyp.12540

[26] Yilmazer C Boccuni L, *et al.*: Effectiveness of somatosensory interventions on somatosensory, motor and functional outcomes in the upper limb post-stroke: a systematic review and meta-analysis. Neurorehabilit. 2019; 44: 459–477. 10.3233/NRE-192687 31256086

[27] Laufer Y Elboim-Gabyzon M: Does sensory transcutaneous electrical stimulation enhance motor recovery following a stroke? a systematic review. Neurorehabil Neural Repair. 2011; 25: 799–809. 21746874 10.1177/1545968310397205

[28] Grant VM Gibson A, *et al.*: Somatosensory stimulation to improve hand and upper limb function after stroke—a systematic review with meta-analyses. Top Stroke Rehabil. 2018; 25: 150–160. 29050540 10.1080/10749357.2017.1389054

[29] Sharififar S Shuster JJ, *et al.*: Adding electrical stimulation during standard rehabilitation after stroke to improve motor function. A systematic review and meta-analysis. Ann Phys Rehabil Med. 2018; 61: 339–344. 29958963 10.1016/j.rehab.2018.06.005

[30] Pan L-LH Yang W-W, *et al.*: Effects of 8-week sensory electrical stimulation combined with motor training on EEG-EMG coherence and motor function in individuals with stroke. Sci Rep. 2018; 8: 9217. 29907780 10.1038/s41598-018-27553-4 PMC6003966

[31] Wymbs NF Bastian AJ, *et al.*: Motor skills are strengthened through reconsolidation. Curr Biol. 2016; 26: 338–343. 26832444 10.1016/j.cub.2015.11.066 PMC4747782

[32] Charalambous CC Alcantara CC, *et al.*: A single exercise bout and locomotor learning after stroke: physiological, behavioural, and computational outcomes. J Physiol. 2018; 596: 1999–2016. 29569729 10.1113/JP275881 PMC5978382

[33] Souron R Besson T, *et al.*: Acute and chronic neuromuscular adaptations to local vibration training. Eur J Appl Physiol. 2017; 117: 1939–1964. 28766150 10.1007/s00421-017-3688-8

[34] Rocchi L Suppa A, *et al.*: Plasticity induced in the human spinal cord by focal muscle vibration. Front Neurol. 2018; 9: 935. 30450077 10.3389/fneur.2018.00935 PMC6225532

[35] Roll JP Vedel J: Kinaesthetic role of muscle afferents in man, studied by tendon vibration and microneurography. Exp Brain Res. 1982; 47: 177–190. 6214420 10.1007/BF00239377

[36] Roll J-P Vedel J, *et al.*: Alteration of proprioceptive messages induced by tendon vibration in man: a microneurographic study. Exp Brain Res. 1989; 76: 213–222. 2753103 10.1007/BF00253639

[37] Burke D: Muscle spindle function during movement. Trends Neurosci. 1980; 3: 251–253.

[38] Seizova-Cajic T Smith JL, *et al.*: Proprioceptive movement illusions due to prolonged stimulation: reversals and aftereffects. PLoS One. 2007; 2: e1037. 17940601 10.1371/journal.pone.0001037 PMC2001181

[39] Goodwin GM McCloskey DI, *et al.*: Proprioceptive illusions induced by muscle vibration: contribution by muscle spindles to perception? Science. 1972; 175: 1382–1384. 4258209 10.1126/science.175.4028.1382

[40] Souron R Baudry S, *et al.*: Vibration-induced depression in spinal loop excitability revisited. J Physiol. 2019; 597: 5179–5193. 31429066 10.1113/JP278469

[41] Nito M Yoshimoto T, *et al.*: Vibration decreases the responsiveness of Ia afferents and spinal motoneurons in humans. J Neurophysiol. 2021; 126: 1137–1147. 34495775 10.1152/jn.00168.2021

[42] Pfenninger C Grosboillot N, *et al.*: Effects of prolonged local vibration superimposed to muscle contraction on motoneuronal and cortical excitability. Front Physiol. 2023; 14: 1106387. 36711014 10.3389/fphys.2023.1106387 PMC9877338

[43] Pettorossi VE Panichi R, *et al.*: Long-lasting effects of neck muscle vibration and contraction on self-motion perception of vestibular origin. Clin Neurophysiol. 2015; 126: 1886–1900. 25812729 10.1016/j.clinph.2015.02.057

[44] Macerollo A Palmer C, *et al.*: High-frequency peripheral vibration decreases completion time on a number of motor tasks. Eur J Neurosci. 2018; 48: 1789–1802. 29923362 10.1111/ejn.14050 PMC6175240

[45] Rabini A De Sire A, *et al.*: Effects of focal muscle vibration on physical functioning in patients with knee osteoarthritis: a randomized controlled trial. Eur J Phys Rehabil Med. 2015; 51: 513–520. 25990196

[46] Pazzaglia C Camerota F, *et al.*: Efficacy of focal mechanic vibration treatment on balance in Charcot-Marie-Tooth 1A disease: a pilot study. J Neurol. 2016; 263: 1434–1441. 27177999 10.1007/s00415-016-8157-5

[47] Pietrangelo T Mancinelli R, *et al.*: Effects of local vibrations on skeletal muscle trophism in elderly people: mechanical, cellular, and molecular events. Int J Mol Med. 2009; 24: 503–512. 19724891 10.3892/ijmm_00000259

[48] Lapole T Pérot C: Effects of repeated achilles tendon vibration on triceps surae force production. J Electromyogr Kinesiol. 2010; 20: 648–654. 20223682 10.1016/j.jelekin.2010.02.001

[49] Souron R Farabet A, *et al.*: Eight weeks of local vibration training increases dorsiflexor muscle cortical voluntary activation. J Appl Physiol. 2017; 122: 1504–1515. 28385918 10.1152/japplphysiol.00793.2016

[50] Holmes MD Vindigni D, *et al.*: What are the temporal and physical characteristics of locally applied vibration that modulate balance in older adults?-A systematic review of the literature. Gait Posture. 2024; 111: 75–91. 38657476 10.1016/j.gaitpost.2024.04.011

[51] Rippetoe J Wang H, *et al.*: Improvement of gait after 4 weeks of wearable focal muscle vibration therapy for individuals with diabetic peripheral neuropathy. J Clin Med. 2020; 9: 3767. 33266464 10.3390/jcm9113767 PMC7700661

[52] Carrasco VB Vidal JM, *et al.*: Vibration motor stimulation device in smart leggings that promotes motor performance in older people. Med Biol Eng Comput. 2023; 61: 635–649. 36574174 10.1007/s11517-022-02733-7

[53] Goebel R Haddad M, *et al.*: Does combined strength training and local vibration improve isometric maximum force? A pilot study. Muscles Ligaments Tendons J. 2017; 7: 186–191. 28717628 10.11138/mltj/2017.7.1.186 PMC5505588

